# Giant Cell Tumor in Tarsal Midfoot Bones: A Case Report

**DOI:** 10.7759/cureus.56215

**Published:** 2024-03-15

**Authors:** Waheeb Abed Alharbi, Hatim Mohammed Alshareef, Yasser B Hennawi, Abdulaziz A Munshi, Abdullah Khalid Alzahrani

**Affiliations:** 1 Department of Orthopedics, King Fahad Armed Forces Hospital, Jeddah, SAU; 2 Department of Orthopedic Surgery, King Fahad Armed Forces Hospital, Jeddah, SAU; 3 College of Medicine, Umm Al-Qura University, Makkah, SAU

**Keywords:** case report, midfoot, pvns, gct, tenosynovial giant cell tumor

## Abstract

Diffuse tenosynovial giant cell tumor (D-TGCT), previously known as pigmented villonodular synovitis (PVNS), is a benign, aggressive, and distracting proliferative synovial lesion. D-TGCT is commonly seen in large joints such as the knee and hip. We present the case of a 57-year-old female who initially presented with swelling on the left midfoot that increased over four years. Clinically, a ganglion was suspected on the left midfoot and an MRI showed a heterogeneous lobulated soft tissue mass on the superior aspect of the tarsal midfoot measuring 5.8 x 2.4 x 4.2 cm. The mass causing remodeling and bony erosion was more appreciated at the medial aspect of the talus bone and extended to the sinus tarsi and talocalcaneal joint space. Surgical excision of the mass was performed, and pathology reports found lobulated soft tissue lesions composed of mononuclear cells, multinucleated giant cells, sheets of foamy macrophages, inflammatory cells, and hemosiderin-laden macrophages. This case represents D-TGCT without atypia or malignancy based on the findings.

## Introduction

Tenosynovial giant cell tumors (TGCT) are rare, benign proliferative synovial lesions that usually affect joints, tendon sheaths, and bursae [1]. There are two types of TGCT: localized (L-TGCT) and diffuse (D-TGCT), which was previously known as pigmented villonodular synovitis (PVNS) [2]. Macroscopically, there are no clear differences between the two types of TGCT. However, magnetic resonance imaging (MRI) can differentiate between these types [1,3]. Moreover, D-TGCT is more aggressive and distracting than L-TGCT from a clinical perspective and is frequently seen in large joints such as the knee and hip [2,4]. In general, TGCT usually presents with pain, swelling, stiffness, and reduced range of motion [5]. Surgical excision is the recommended treatment for both types of TGCT [6]. Although there are various surgical approaches, all have a noticeable recurrence rate [7]. We report a case of a 57-year-old female with a left midfoot D-TGCT. It is important for physicians to be aware of the diagnostic challenges associated with D-TGCT and the best diagnostic modality when suspecting D-TGCT.

## Case presentation

The 57-year-old female was not known to have any medical illnesses and complained of left midfoot dorsal swelling for the past four years, with no history of trauma and no constitutional symptoms. The swelling increased with time. She had a normal alignment of the left ankle and foot, with mild swelling of the ankle, hindfoot, and midfoot. There were no points of tenderness around the ankle joint. Dorsally, there is a mass around 5 x 5 cm with palpable, soft, mobile swelling with bruising over the swelling. She had palpable dorsalis pedis. Clinically, a ganglion was suspected on the left midfoot. X-rays showed no abnormalities.

**Figure 1:**
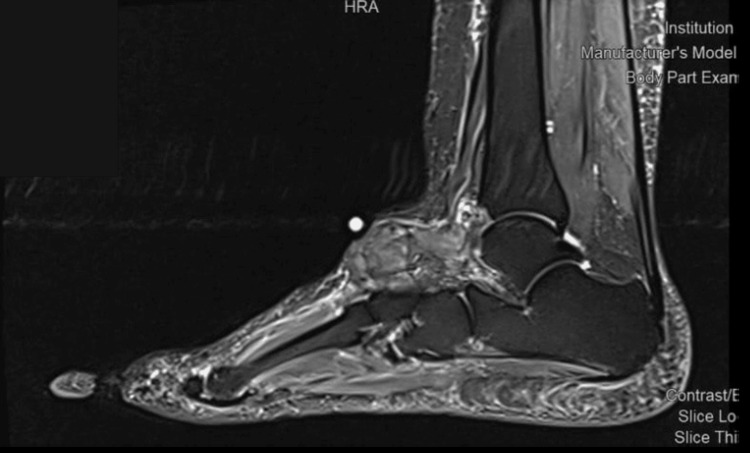
Preoperative magnetic resonance imaging study of the left foot lateral view showing heterogeneous lobulated soft tissue mass at the superior aspect of the tarsal midfoot bones.

**Figure 2:**
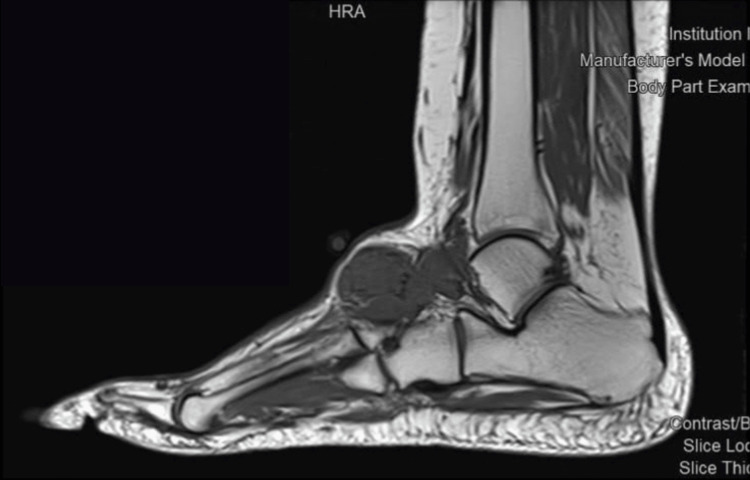
Preoperative magnetic resonance imaging study of the left foot lateral view showing heterogeneous lobulated soft tissue mass at the superior aspect of the tarsal midfoot bones.

**Figure 3:**
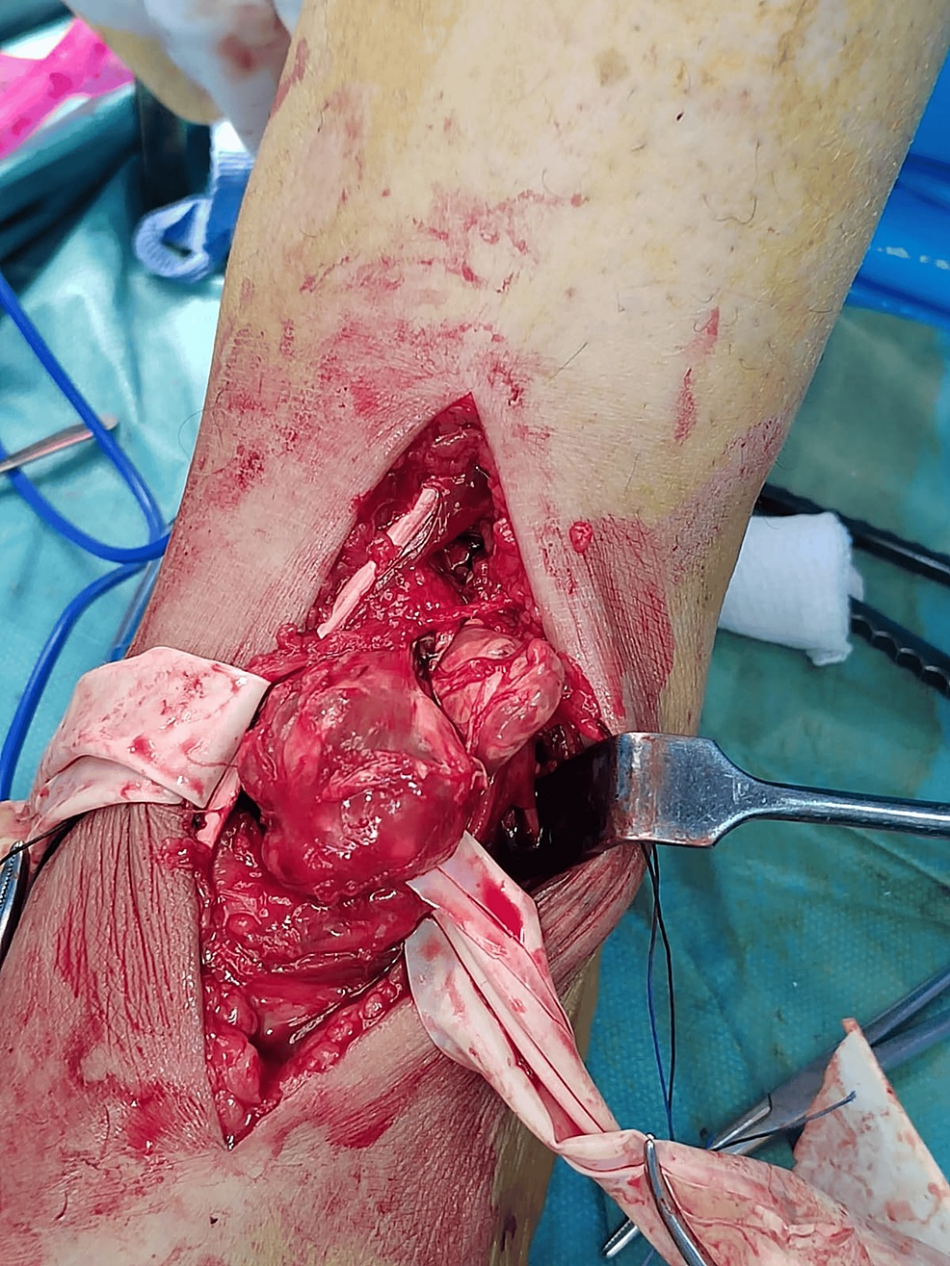
Intraoperative photograph of mass excision

The MRI showed heterogeneous lobulated soft tissue mass at the superior aspect of the tarsal midfoot bones, measuring 5.8 x 2.4 x 4.2 cm. The mass displaced and encased the adjacent anterior ankle tendons. Multiple degenerative cystic changes were noted at the tarsal bones, predominantly at the navicular and talus bones. There were degenerative changes of the foot prominently at the metatarsal pharyngeal joint and proximal interphalangeal manifested in subchondral cystic degenerative charges with synovial thickening and minimal joint effusion. The mass causing remodeling and bony erosion was more appreciated at the medial aspect of the talus bone and extended to the sinus tarsi and talocalcaneal joint space (Figures 1-2). These findings were suggestive of PVS. Another possible differential, such as TGCT or other pathology, could not be totally excluded for histopathology correlation. A surgical excision of the mass was performed, and a sample was taken to the pathology (Figure 3), which showed lobulated soft tissue lesions composed of mononuclear cells, multinucleated giant cells, sheets of foamy macrophages, inflammatory cells, and hemosiderin-laden macrophages. Cleft-like spaces and foci of stromal hyalinization were present. No mitosis or necrosis was seen. Furthermore, immunohistochemistry was performed, with all controls showing appropriate reactivity.

The target cells were positive for CD 68 and the target cells were negative for desmin and smooth muscle action (SMA). It was concluded that this was a case of D-TGCT without atypia or malignancy.

## Discussion

D-TGCT, also called PVNS, is a subtype of TGCT that primarily affects the synovium, often involving joints, bursae, and tendon sheaths [8]. Localization in the foot is relatively rare compared to other parts, like large joints and tendinous sheaths of the fingers. Previously, various authors have reported the incidence ranging from 11% to 20% of PVNS in the foot [9,10]. We present a case of a 57-year-old female with midfoot swelling that was initially misdiagnosed as a ganglion but later revealed to be PVNS through histopathological, imaging, and clinical evaluations. This case presentation highlights the diagnostic challenges associated with PVNS. In our case, the patient reported midfoot dorsal swelling, which increased progressively over four years. These findings are supported by various previous studies, which confirm that PVNS is a slow-growing lump [11,12]. In most cases, the lump is painless. However, pain has been reported in rearfoot parts where the affected bone is involved [13]. Initially, the clinical presentation and X-ray findings suggested a ganglion, a relatively benign soft tissue mass commonly encountered in clinical practice. In the context of giant cell tumors, the use of X-ray imaging is considered essential. It has been found that approximately 33% of cases exhibit bone abnormalities [14]. Standard radiographs often reveal various radiological irregularities, which may include bone erosions, degenerative lesions, or the presence of soft tissue masses. Notably, cystic erosions are more commonly observed and indicative of the condition, particularly in the hand and hip regions [15].

In this case, histopathological and MRI findings were critical in establishing the correct diagnosis of PVNS. This aligns with previous studies as well, which have shown the efficacy of MRI in the diagnosis of PVNS [16,17]. The MRI results of PVNS may exhibit similarities with other synovial lesions [18]. On T1-weighted images, the lesions have a signal intensity that is either low or intermediate. PVNS exhibits diminished signal intensity on T2-weighted and gradient-echo imaging. The hypothesis posits that the presence of hemosiderin in PVNS is responsible for the observed low signal intensity on T2-weighted imaging [19]. In this case, septations and signal void foci are consistent with the typical MRI appearance of PVNS [20]. Moreover, the mass's erosive effects on the adjacent bones and the displacement of nearby tendons were indicative of the locally aggressive nature of PVNS.

The establishment of a correlation between the histologic characteristics and the imaging results is crucial in order to accurately determine the diagnosis of a benign lesion associated with a diffuse synovial process. Histologically, PVNS is characterized by a proliferation of synovial-like cells, multinucleated giant cells, macrophages, and hemosiderin-laden macrophages within the joint synovium [3]. The macroscopic pathological presentation of PVNS typically manifests as an infiltrative neoplasm that extensively affects and increases the thickness of the synovial membrane over the whole joint. The structure of synovial hypertrophy is characterized by the presence of irregular papillary or villous projections, as well as bigger nodular or villonodular protrusions [3]. The presence of these characteristic features, along with immunohistochemical staining results, confirmed the diagnosis in this case. Immunohistochemistry, particularly the positive staining for CD68 and negative staining for desmin and SMA, helped rule out other potential diagnoses [21].

According to the existing literature, PVNS is rarely observed in the foot and its etiology remains unknown. However, several theories regarding its etiology, such as an inflammatory process, neoplasia, or lipid metabolism issue, are frequently discussed and subject to ongoing dispute [3]. The primary approach for managing this tumor is predominantly surgical, given the documented instances of malignant progression subsequent to radiotherapy [22]. Historically, the initial approach for treatment is curettage, either with or without the addition of bone grafting. While often efficacious, it has been correlated with a significantly elevated recurrence rate ranging from 15% to 60%. Recurrence is frequently observed to manifest within a span of three years following the initial surgical intervention, and is commonly regarded as a chronic condition rather than a mere relapse [23].

## Conclusions

There are very few cases of PVNS affecting the tarsal bones. History and clinical examination along with investigation, particularly an MRI scan, is the best method. It is important to perform a complete excision and close follow-up to minimize the high risk of recurrence.
